# Percentages and Absolute Numbers of CD4+CD8+ Doublepositive T Lymphocytes in the Peripheral Blood of Normal Italian Subjects: Relationship with Age and Sex

**DOI:** 10.4274/tjh.galenos.2019.2019.0452

**Published:** 2020-05-06

**Authors:** Alessandra Marini, Daniela Avino, Monica De Donno, Francesca Romano, Riccardo Morganti

**Affiliations:** 1 Laboratory of Clinical Pathology, Versilia Hospital, Lido di Camaiore, Italy; 2 Unit of Hematological Diagnostics, A. Tortora Hospital, Pagani, Italy; 3 Section of Statistics, AOUP, Pisa, Italy

**Keywords:** CD4+CD8+ double-positive T lymphocytes, Flow cytometry

## To the Editor,

We read with great interest the paper by Gonzalez-Mancera et al. [1] concerning the percentages of CD4+CD8+ double-positive T-lymphocytes (DPTs) in normal subjects. DPTs are a small subset of T cells normally found in the peripheral blood. Their functions appear to be controversial, since both cytotoxic and suppressive roles have been reported [2].

The paper by Gonzalez-Mancera et al. [1] assessed the frequency of DPTs in a large cohort of normal subjects. This topic is very interesting, since only a few papers with the aim of establishing reference values of DPTs have been published. Previous studies were carried out with Spanish and German subjects [3,4], while that of Gonzalez-Mancera et al. [1] took Colombian individuals into consideration.

With regards to Italy, to the best of our knowledge, no data about the frequency of DPTs have been produced so far. It is noteworthy that the largest multicenter Italian study, carried out in 1999, did not evaluate DPTs [5].

Therefore, we revised our electronic files on normal Italian subjects referring to our laboratories for routine controls. We evaluated 238 subjects (males=84; females=154) with normal complete blood counts and hematochemical values. Flow cytometry was carried out with a FACSCanto II cytometer, assisted by FACSCanto software. A single platform assay was performed using the BD Multitest 6-color TBNK reagent and Trucount tubes. All subjects showed normal percentages and absolute counts of CD3+, CD4+, CD8+, CD19+, and CD16/CD56+ lymphocytes. The CD4:CD8 ratio was always >1. Percentages and absolute counts of CD4+CD8+ DPTs were calculated by automated lymphocyte gating.

Continuous data were described by mean, standard deviation (SD), median, and interquartile range. Comparisons between CD4+CD8+ DPTs and age categories or sex were performed by two-way ANOVA followed by multiple comparisons (LSD method). Significance was fixed at 0.05. All analyses were carried out with SPSS 25.

Results are shown in Tables 1 and 2 and are expressed both as percentages and absolute counts. We found that the comparisons of DPTs with the factors of “sex” and “sex-age” were not significant (p=0.533 and p=0.398, respectively). Interestingly, we found a statistically significant increase of DPTs with age. This phenomenon was more evident when younger subjects (especially 20-30 years old) and older subjects (older than 50 years) were compared.

Previous studies showed discordant results, since DPT frequency was found to increase with age in Spanish individuals [3] but to decrease with age in German males [4]. These two studies did not find a relationship between DPT frequency and sex, in agreement with our results. On the contrary, Gonzalez-Mancera et al. [1] reported that women showed a significantly higher DPT percentage than males.

Our method did not allow us to make a distinction between CD4^high^CD8^low^ and CD4^low^CD8^high^, as done by Gonzalez-Mancera et al. [1]. Nevertheless, we think that our study might provide some novel information about reference values of DPTs and might encourage further studies, since this subset of lymphocytes might play a significant role in some human diseases.

## Figures and Tables

**Table 1:**
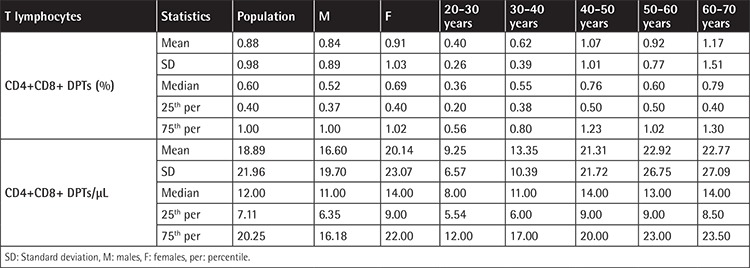
Descriptive analysis of double-positive T lymphocytes (DPTs), stratified for sex and age. The comparisons of DPTs with the factors “sex” and “sex-age” are not significant (p=0.533 and p=0.398, respectively).

**Table 2:**
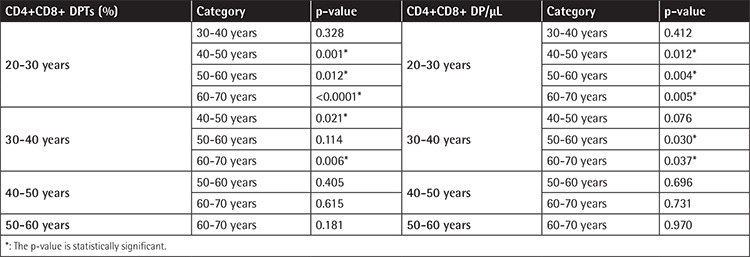
Inferential analysis of DPTs: multiple comparisons related to age categories after two-way ANOVA (age and sex).
